# Biomechanical evaluation of an experimental internal ring fixator (RingFix) for stabilization of pelvic ring injuries on an osteoporotic bone model

**DOI:** 10.1038/s41598-024-71138-3

**Published:** 2024-09-06

**Authors:** Charlotte Arand, Christian Hartung, Dorothea Mehler, Erol Gercek, Jochen Wollstädter, Daniel Wagner, Pol M. Rommens

**Affiliations:** 1 Department of Orthopaedics and Traumatology, University Medical Center, Langenbeckstraße 1, 55131 Mainz, Germany; 2 Departement of Orthopedics and Tramatology, Lausanne University Hospital, Rue du Bugnon 46, 1011 Lausanne, Switzerland

**Keywords:** Pelvic ring fracture, Insufficiency fracture, Ring fixator, Minimally-invasive fracture fixation, Bone, Trauma, Fracture repair

## Abstract

During the last decades, effective pain reduction and early mobilization were identified as the central priorities in therapy of insufficiency fractures of the pelvis. For operative treatment minimally-invasive stabilization techniques are favored. While there is consensus on the significance of sufficient dorsal stabilization the role of additional fixation of the anterior fracture component stays under discussion. Within the present study we developed an internal ring fixator system (RingFix) with the question whether an in-itself-closed construct can improve stability of the entire ring structure. RingFix was evaluated on an osteoporotic bone model with a standardized FFP IIIc fracture within an established biomechanical setup regarding its primary stabilization potential. Further, it was compared to transiliac–transsacral screw fixation with and without stabilization of the anterior fracture component. The transiliac–transsacral fixation with separate screw fixation of the anterior fracture showed significantly higher stability than the RingFix and the transiliac–transsacral screw fixation without anterior stabilization. Our results show that stabilization of the anterior fracture component relevantly improves the stability of the entire ring construct. As a bridging stabilizer, RingFix shows biomechanical advantages over an isolated dorsal fracture fixation, but inferior results than direct stabilization of the single fracture components.

## Introduction

The observed increasing incidence of fragility fractures of the pelvis (FFP) led to a special interest in these pathologies over the last decade and opened a wide field of research^1,2^. With the successful implementation of specific classification systems, treatment recommendations and algorithms were contrived^3–5^. The central objective in treatment of affected, typically frail patients is a sufficient pain control to allow for early remobilization. Analgesic therapy and physiotherapeutic mobilization is regarded as the primary therapy in isolated anterior (FFP I) and non-displaced posterior fractures (FFP II)^3^. In patients with posteriorly displaced fractures (FFP III and FFP IV) or with prolonged pain and thereof immobilization, surgical treatment is recommended^6^. The objective of surgical intervention in those patients is not an anatomical reduction but a stable in-situ fixation to achieve sufficient pain control and to allow for immediate postoperative remobilization of the patient. Further, the surgical therapy should be as minimally-invasive as possible to minimize surgically-related complications in this fragile patient group with commonly reduced bone quality. While there is consensus about the necessity of a stable fixation of the posterior pelvic ring, discussion remains controversial about indications and methods for stabilization of the anterior fracture part.

For minimal-invasive stabilization of the posterior pelvic ring in FFP, different percutaneous (trans-) iliosacral^7–13^ and transiliac internal fixation techniques^14,15^ are described. The anterior fracture component is often left without fixation^2,16^. However, the placement of an internal^17,18^ or external fixator^19^, percutaneous screw fixation of the superior pubic ramus^16,20,21^, or open reduction and plate osteosynthesis^22^ are common methods used for surgical stabilization of anterior fracture components with overall good results reported but also a relevant number of implant related complications like implant loosening or backing out of screws^22,23^. Within a biomechanical study on an FFP Type IIIc fracture in an osteoporotic pelvic bone model we could show, that the stabilization of the anterior fracture component in addition to a trans-iliosacral posterior fixation enhances the stability of the entire ring construct significantly^16^. These results raised the question whether an in-itself-closed ring fixation construct augmenting the pelvic ring might provide higher stability than a separate in-situ fixation of the single fracture components.

Thus, within the present pilot study, we built up an in-itself-closed internal ring fixator for the pelvic ring fixation (RingFix) and evaluated the stability of the RingFix on an FFP Type IIIc fracture in an osteoporotic pelvic bone model within a standardized biomechanical test setup in comparison to transiliac–transsacral screw fixation as an established fixation method with and without fixation of the anterior fracture component.

## Material and methods

The test setup and the utilized bone models were similar as in a biomechanical study on FFP Type IIIc fractures recently published by our group^16^.

For the actual study, 24 explicit osteoporotic synthetic bone models (‘Sawbone Type 1301-1’, Fa. Sawbones, Sawbones Europe AB Servicing Europe, Middle East, and Africa, Malmö, Sweden) were used and subdivided into three groups with different fixation methods.

To simulate a standardized FFP Type IIIc fracture, in all bone models a complete fracture of the sacral ala was generated on the left side lateral to the neuroforamina using a 3D printed sawing template. A standardized vertical-oblique fracture of the superior and inferior pubic ramus was generated also on the left side in the area of the obturator foramen, consistent with a Nakatani II fracture^23^. In all samples a 3 mm thick felt was placed into the fracture gap to simulate a partially unstable fracture.

To achieve optimum comparability standardized position of the implants was achieved by using customized 3D printed drilling templates which were calculated previously on the CAD data set of the bone model. Eight of the bone models received a trans-iliosacral screw fixation with a cannulated 7.3 mm semi-threaded trans-iliosacral screw (140/32 mm) with washer in S1 and a shorter, oblique 7.3 mm fully-threaded iliosacral screw with washer in S1 ending up in the area of the promontory. Further eight bone models received the same stabilization with an additional cannulated 7.3 mm semi-threaded retrograde transpubic screw for fixation of the anterior pelvic ring.

**Fig. 1:**
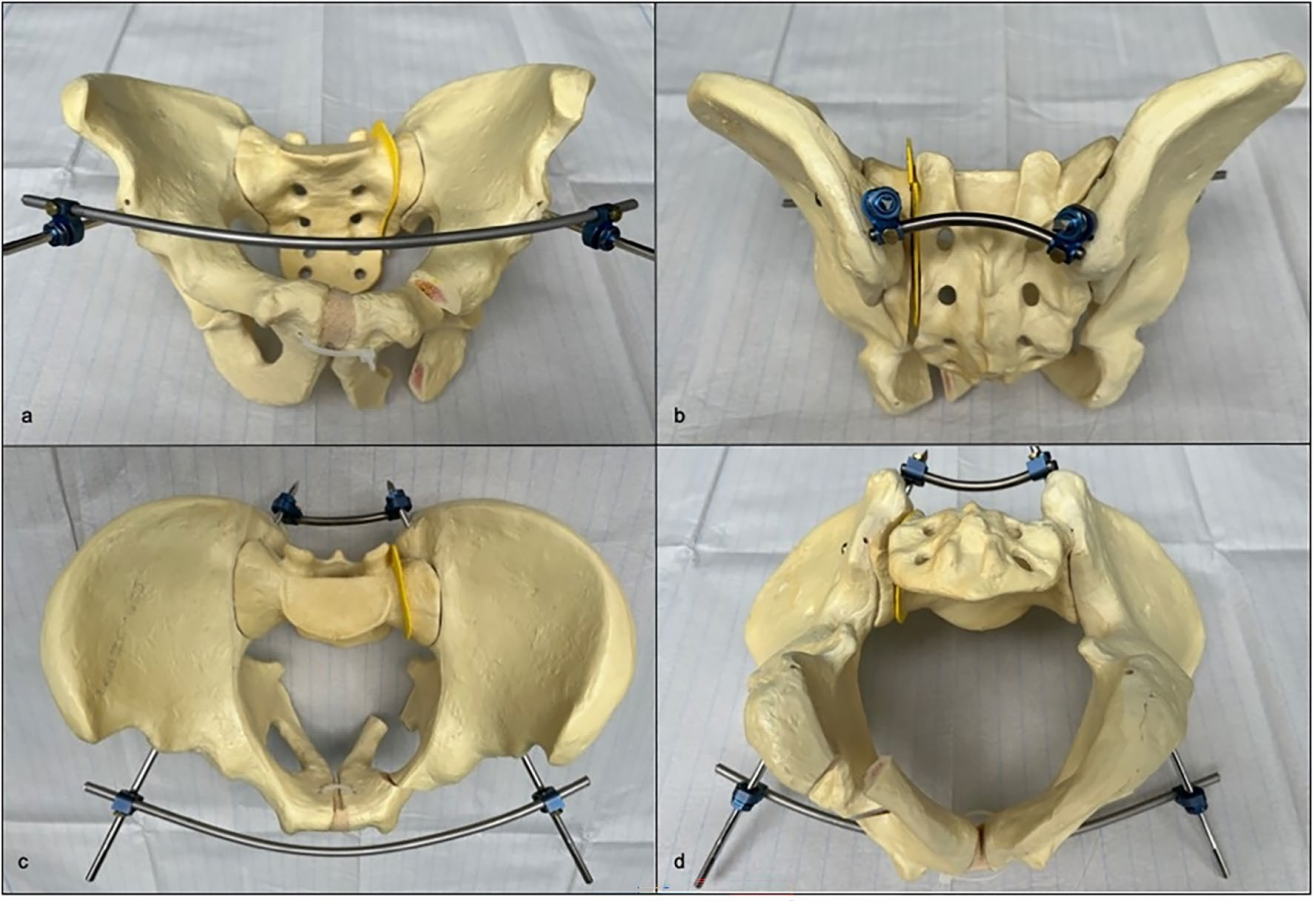
Synthetic pelvic bone model with a standardized FFP type IIIc fracture refixated with RingFix in (**a**) ap, (**b**) pa, (**c**) inlet and (**d**) a reversed-inlet caudal perspective.

The remaining eight bone models were stabilized with the RingFix. RingFix was built up on two 5.0 mm Steinmann pins with central thread introduced slightly lateral to the anterior inferior iliac spine, passing the ilium completely intraosseous and ending up after passing the posterior cortex of the ilium in the area of the posterior superior iliac spine. Right and left Steinmann pins were connected posteriorly over a pre-bent 5.0 mm rod as used for dorsal instrumentation in spine surgery and the specific connector clamps for Steinmann pin-rod-connection of the USS II system of DePuy Synthes (USS Universal Spine System, DePuy Synthes, Johnson & Johnson, New Brunswick, New Jersey, USA) (Fig. 1).

After posterior connection, reduction of the anterior fracture part was performed by connecting the anterior ends of the Steinmann pins via another pre-bent 5.0 mm rod and clamps under slight adduction force. The posterior and anterior rods are meant to be located subcutaneously, pins could be introduced in anterior–posterior direction as performed in the lab as well as in posterior-anterior way.

As all peripelvic ligaments are missing within this model, an additive iliosacral screw was used within the RingFix group to compensate the lack of the dorsal ligamentous apparatus and achieve a comparable rotational stability in all three groups. For this propose an additional short oblique 7.3 mm fully-threaded iliosacral screw with washer in S1 ending up in the area of the promontory was introduced following the same screw corridor as in the two other groups.

Groups were named as: trans-iliosacral and iliosacral screw with anterior fixation (SI +), trans-iliosacral and iliosacral screw without anterior fixation (SI−); ring fixator with iliosacral screw (RingFix).

**Fig. 2:**
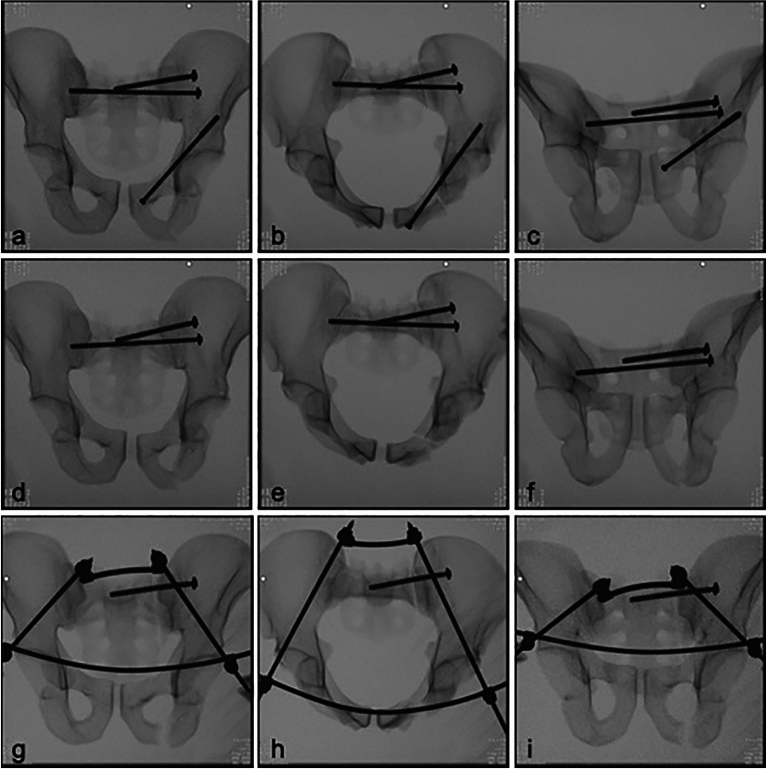
X-ray imaging of implant position in the SI-screw fixation group with anterior fixation (SI +) (**a**–**c**) and in the SI-screw fixation group without anterior fixation (SI−) (**d**–**f**) and the ring fixator group (RingFix) (**g**–**i**) in ap (**a**, **d**, **g**), inlet (**b**, **e**, **h**) and outlet-projection (**c**, **f**, **i**).

All bones were checked radiographically in ap, inlet and outlet view for correct implant positioning after stabilization (Fig. 2).

**Fig. 3:**
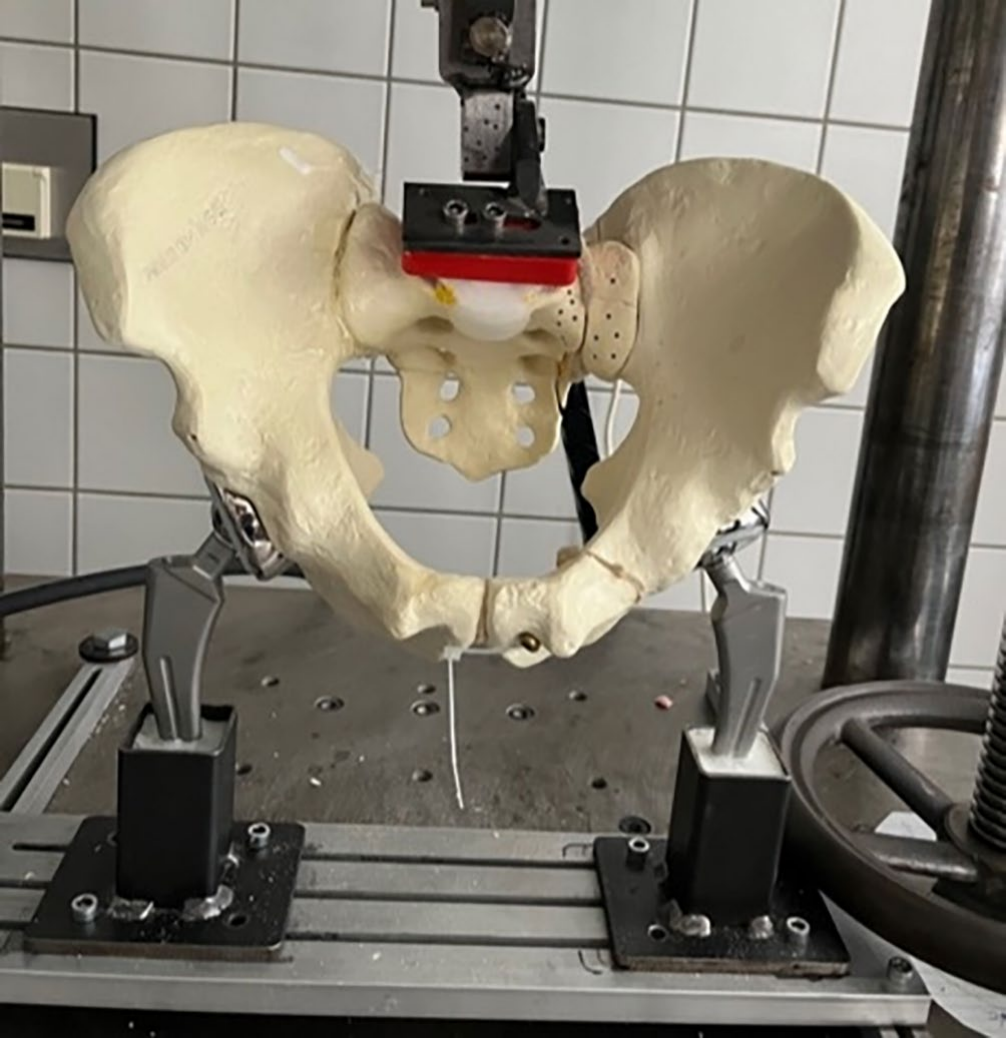
Sawbone specimen (group SI +) set ob two bipolar hip prothesis and attached to the lever arm of the test machine at the endplate of S1.

Testing was performed on a servo-pneumatic test machine (Fa. SincoTec, Clausthal-Zellerfeld, Germany). The bones were attached to the lever arm of the test machine over a small PMMA block screwed to the endplate of S1 simulating the loading transmission from the lumbar spine to the sacrum, and set on to two fixed bipolar hip protheses building up a counterfort, simulating a standing position with a pelvic tilt of about 15°. In this physiological position axial loading could be performed with a minimum of shear forces. Further details of the test setup are described in a previous study of our group^16^. Figure 3 shows a picture of the test setup with a SI + specimen attached to the test machine.

As the pelvic ring is loaded with up to two to three times the body weight during standing and normal walking force-controlled axial loading was performed with a pre-load of 50 N followed by cyclic loading with 50–1200 N for a total of 1000 test cycles with a frequency of 0.05 Hz, in order to simulate a two-legged stance^24,25^. The translation of the lever arm that was needed to generate the demanded forces was measured. Testing was interrupted and finished early in case of breakdown of the bone, or if the measured translation of the level arm exceeded 25 mm according to the existing test protocol^16^. The number of cyclic axial loading at failure was documented for each pelvic model. Optical tracking of fracture sites was performed separately for the anterior and the dorsal fracture component using a camera system and the Software Simi Motion (Simi Motion 2D/3D, Fa. Simi Reality Motion Systems GmbH, Unterschleißheim, Germany, www.simi.com) observing movements on the fracture sites based on in total eight optical markers. Statistical evaluation was performed using GraphPad Prism (Prism 9.5.1, GraphPad Software, 225 Franklin Street. Fl. 26, Boston, MA 02110, USA, www.graphpad.com) applying Mann–Whitney-U test and Kruskal–Wallis test.

## Results

**Fig. 4:**
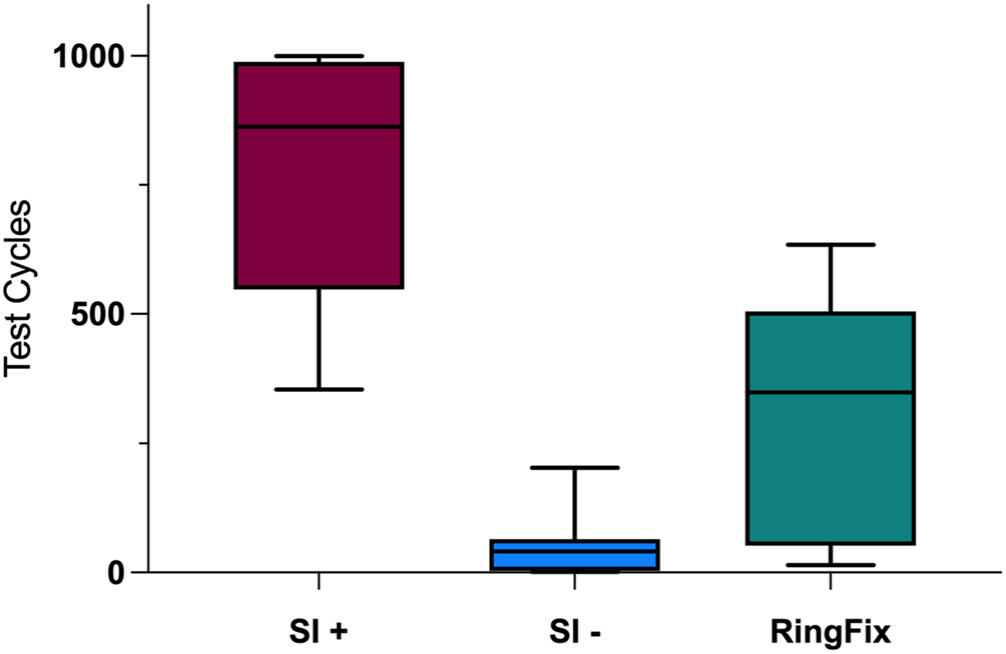
Graphic overview on the reached loading cycles. SI + , SI-screws with anterior fixation, SI−, SI-screws without anterior fixation; RingFix, ring fixator. Comparison show statistical significant differences: SI + vs. SI−: *p* = 0.0002; SI + vs. RingFix: *p* = 0.0047; SI− vs. RingFix: *p* = 0.0274.

Only two of the 24 bones finished 1000 axial loading cycles according to the protocol. In the SI + group, two of eight bones finished the test. In the SI− and in the RingFix group none of the bones finished the total of 1000 loading cycles. The SI + group showed the highest stability (median of reached cycles 862.5), followed by the RingFix group (median of reached cycles 349.0) and the SI− (median of reached cycles 41.0). Table 1 shows the reached number of loading cycles at failure, Fig. 4 gives a graphic overview on the number of reached loading cycles.

**Table 1:** Reached number of test cycles of each single bone.

	SI +	SI−	RingFix
1	1000	46*	14*
2	355*	9*	400*
3	900*	1*	323*
4	953*	67*	375*
5	542*	1*	634*
6	825*	203*	33*
7	1000	58*	540*
8	565*	36*	108*
Median (range)	862.5 (645)	41.0 (202)	349.0 (620)
percentile 25%/75%	547.5/988.3	3.0/64.8	51.6/505.0

SI + , SI-screws with anterior fixation, SI−, SI-screws without anterior fixation; RingFix, ring fixator. *Marks the occurrence of a new fracture.

**Fig. 5:**
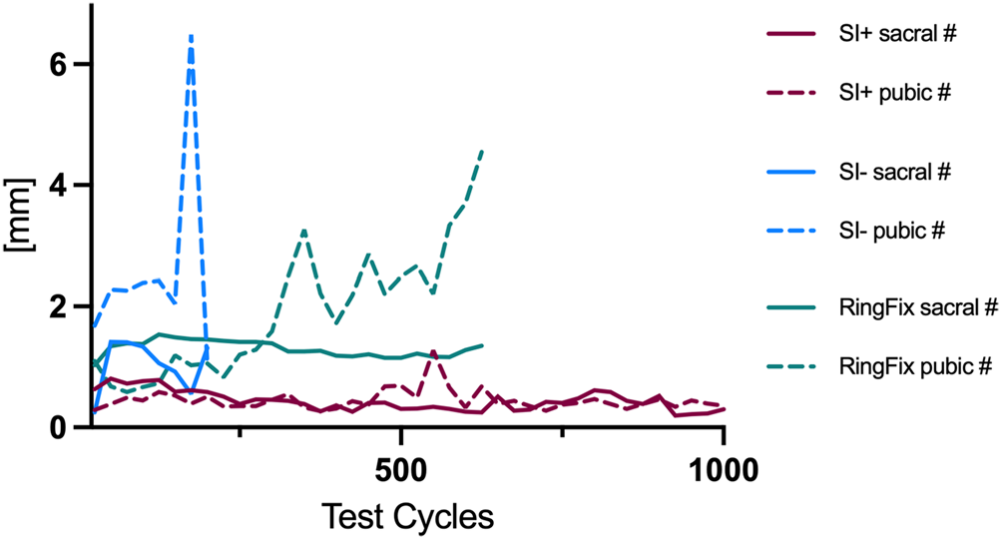
Observed interfragmentary movement at the fracture sites under cyclic loading throughout the testing. Interfragmentary movement was observed to be significantly less within the SI + group compared to SI− and RingFix o both fractures sites (SI + vs. SI− *p* < 0.001; SI + vs. RingFix *p* < 0.001; SI− vs. RingFix *p* = 0.7263).

Optical measurements showed a heterogenic pattern throughout the testing. While within the SI + group a rather consistent movement at the fracture sites was observed, the SI− and RingFix group showed an increasing movement at the fracture gap especially of the pubic fracture. Figure 5 illustrates the median measured movements at the fracture site separately for the sacral and the pubic fracture.

As within the SI− and RingFix group none of the specimen finished the test according to the protocol the respective curve ends early.

Furthermore, we observed new sacral fractures on the contralateral side of the initial fracture. Those new fractures occurred more frequently and earlier during testing in the bone models without anterior fixation.

## Discussion

In this study we built up an in-itself closed ring fixator (RingFix) for internal stabilization of pelvic ring fractures. To explore its mechanical properties and therapeutic potential, we performed a biomechanical evaluation of the RingFix on a standardized an FFP Type IIIc fracture in an explicit osteoporotic sawbone model. We observed a significantly lower stability after bridging fixation with the in-itself closed internal pelvic ring fixator (RingFix) than after direct stabilization of all individual fracture components, but a significantly higher stability than after intra-osseous trans-sacral fixation without fixation of the anterior fracture component. Especially at the pubic fracture gap more and increasing movement over time was observed within the groups without direct anterior fracture fixation. The results show that the RingFix system has no advantages as a stand-alone implant for the fixation of FFP Type IIIc fractures or fracture types with a higher instability over existing fixation methods, but point out again the impact of an anterior fixation to the hole construct’s stability.

Discussions on when and how to operate and the question whether both the posterior and anterior pelvic ring should be stabilized are still open and a standardized treatment protocol for FFP is lacking. There is a broad consensus that adequate pain control is crucial in all fracture types to allow for early mobilization of the patient and thereby preventing longer or permanent immobility with its associated complications. There is also consensus on the role of surgical treatment for mechanical stabilization of unstable fracture types as well as for extended pain therapy and pain control. Specific classification systems for osteoporotic and insufficiency fractures of the pelvic ring, such as the FFP classification by Rommens et Hofmann^3^ and the OF classification following the corresponding classification system for vertebral body fractures^5^, have led to an enhanced understanding of these fractures and to specific recommendations for an adequate treatment in relation to the fracture type^26^. The stabilization of the posterior pelvic ring is accepted as the primary goal in the operative treatment of FFP. Different minimally-invasive alternatives are available. As the most common method different intra-osseous sacroiliac and transiliac–transsacral fixation techniques are described^1,2,7–10,12^. Cintean et al. showed within a biomechanical study a significantly reduced interfragmentary movement in FFP Type IIc fractures using a transiliac–transsacral fixation method in comparison with unilateral fixation^27^. Further, Dienstknecht et al. did not find a significant difference in stability between iliosacral screw fixation and transiliac internal fixation (TIFI) of a complete iliosacral separation^28^. El-Hamalawy et al. did not find a difference in clinical outcome between intra- and extra-osseous sacral fixation of posterior pelvic ring injuries in a young patient cohort^29^. Within a finite element analysis Salasek et al. performed a biomechanical comparison of a transiliac internal fixator and two iliosacral screws for treatment of transforaminal sacral fractures and found significantly higher stiffness and lower stress in the group of the TIFI model^30^.

From this data, we may conclude that intra- and extra-osseous bridging fixation of posterior pelvic ring fractures can be considered equivalent. TIFI may be the technique of choice in patients with a dysmorphic sacrum, in which a safe trans-sacral corridor is not available^31,32^.

The necessity of stabilization of the anterior fracture part remains controversial. Several studies report about good clinical and radiological results after posterior without additional anterior stabilization^2,33^. In their biomechanical study, Osterhoff et al. emphasized the role of the pectineal ligament as a relevant secondary stabilizer of the anterior pelvic ring^34^ which might be a reason why conservative treatment of anterior pelvic ring fractures is successful in fractures without relevant dislocation and thereof still intact ligaments. Nevertheless, it remains unclear how the integrity of the anterior ligaments can be ensured in clinical practice.

On the other hand, several authors report about good treatment results and major pain control addressing the anterior fracture component as well. Several minimally invasive techniques are in practice. The supraacetabular external fixator uses the anterior part of the supraacetabular corridor for the placement of its Steinmann pins over small incisions. However, a relatively high complication rate is associated with this method including pin track infection, wound breakdown, and difficult mobilization of the patient due to extensive hardware^19,20,35^. Internal fixator (INFIX) systems consisting of a subcutaneous, curved rod connecting the supra-acetabular fixator pins or screws were developed to provide comparable stability, and to avoid the complications of the external fixator. In addition, Hack et al. showed within a biomechanical study that there was less plastic deformation and higher stiffness found in the internal fixator group as compared to an external fixator group^36^. However, literature data also reveal that the internal fixator is associated with a significant complication rate including periarticular ossification, and damage to the femoral vessels and the femoral and lateral femoral cutaneous nerves^17,18,37,38^. Percutaneous screw fixation of the anterior pelvic ring in antegrade or retrograde direction is another fixation technique. It is described as a sufficient and minimally invasive fixation method for fractures without relevant displacement^20,39^. Even for displaced fractures, retrograde percutaneous screw fixation is described to be possible using a special manoeuvre^21^. Nevertheless, the insertion of a straight 7.3 mm screw is only possible in half of the trans-pubic corridors^40^. Fractures with a relevant displacement or very near to the pubic symphysis are reduced and fixed with open reduction and plate osteosynthesis. In their retrospective analysis of a total of 48 patients with FFP, Herteleer et al. described a high rate of screw loosening with loss of stability^22^.

The objective of the present study was the development and mechanical evaluation of an in-itself closed ring fixator system (RingFix) for internal stabilization of FFP. The RingFix can be regarded as a merger of the TIFI and the INFIX. It closes the broken pelvic ring indirectly and independent of the fracture location. Fixation of both the posterior and anterior pelvic fracture components is achieved without a direct in-situ stabilization of the fractures. The results of this biomechanical study show that an in-situ stabilization of the single fracture components (SI + group) is significantly superior to an indirect (RingFix group) or incomplete (SI− group) fixation.

Thinking of a potential use in clinical practice there are some further concerns to mention. The insertion of the RingFix would require to change the patient’s position from prone to supine at least once during the surgical procedure which might prolong the surgical procedure relevantly. Further, the reported problems and observed complications of the INFIX involving the femoral vessels and nerves at risk would remain as well as soft tissue complications like potential skin irritation and ulceration^18,37,38^.

We conclude that the RingFix cannot be used as a stand-alone implant for the stabilization of an unstable FFP type with a posterior and anterior fracture component as it does not provide any advantages over direct fracture fixation. It might have a role as an additive fixation tool in cases of very poor bone quality or bone defects and could be considered as an option in special situations of traumatic pelvic ring fractures with bone defect or extended soft tissue damage.

There are several limitations of the study to state. All tests were performed on synthetic bone models. Even if a specific osteoporotic bone model was used, the artificial bone model does not fully simulate the individual physiological variability of bone mineral density within the pelvic bone. Further, secondary stabilizers such as muscles and especially strong peripelvic ligaments like the pectineal ligament and the iliolumbar ligaments are missing in this model. For this, an additive iliosacral screw was used within the RingFix group to compensate the lack of the dorsal ligamentous apparatus and achieve a comparable rotational stability in all three groups. As the peripelvic ligaments are missing within our model, construct stability might be underestimated in general within this study. Another limitation to mention is that in our test setup an upright stand with only symmetric axial loading was simulated. Tensile forces and asymmetric loading as they occur during walking were not reproduced.

## Conclusion

Our observations suggest that:

1. An in-itself closed internal ring fixator construct (RingFix) does not show any advantages over in-situ stabilization of the posterior and anterior fracture components of an unstable FFP type.
2. Fixation of the anterior fracture significantly improves the stability of the entire pelvic ring construct.

## Data Availability

Raw data were generated at University Medical Center of Johannes Gutenberg University Mainz, Germany. Derived data supporting the findings of this study are available from the corresponding author [C.A.] on request.
